# Supplementary material for: Outdoor attractive targeted sugar bait Phase III trials for malaria control in Kenya, Mali, and Zambia: An individual participant data meta-analysis
Source: PLOS Glob Public Health. 2026 Oct 9;6(10):e0006513. doi: 10.1371/journal.pgph.0006513 (PMC13652216; doi:10.1371/journal.pgph.0006513)
Supplement: Supplementary file 1 [file pgph.0006513.s001.docx]

**Outdoor attractive targeted sugar bait Phase III trials for malaria control in Kenya, Mali, and Zambia: An individual participant data meta-analysis**

Ruth A. Ashton^1^, Daniel P. McDermott^2^, Fousseyni Kane^3^, Sophie Sarrassat^4^, Angela Harris^5^, Christen Fornadel^5^, Joseph Wagman^6^, Javan Chanda^7^, Megan Littrell^8^, Feiko O. ter Kuile^9,10^, Aaron M. Samuels^10,11^, Eric Ochomo^2,9^, Thomas S. Churcher^12^, Joseph Biggs^4^, Sarah G. Staedke^2,9,10^, Seydou Doumbia^3^, Immo Kleinschmidt^4^, Joshua Yukich^1^, Thomas P. Eisele^1^

**Table A** Effect of ATSB density on malaria clinical incidence among children, when limited to ATSB densities that exist in all three sites (range of shared support). Models include baseline cluster incidence and site as fixed effects, and cluster as random effect.

The range of shared support across the three sites was 6.0-13.2 ATSB/hectare for cluster-level ATSB density, 3.2-12.7 ATSB/hectare for coverage-adjusted cluster-level density, and 2.6-12.7 ATSB/hectare for intact coverage-adjusted cluster-level density. The range of shared support was 1.7-57.3 ATSB/hectare for local ATSB density, and 1.7-57.3 ATSB/hectare for both coverage-adjusted and intact coverage-adjusted local ATSB density.

Cluster-level density was defined as the total ATSB deployed in the cluster divided by the total area of the cluster in hectares. Local density was defined as the number of ATSB deployed in a 1 hectare area surrounding the cohort participant household.

| **Density** | **Population** | **Exposure*** | **N children / clusters** | **IRR** | **95% CI** | **p** |
| --- | --- | --- | --- | --- | --- | --- |
| Cluster-level | Both arms, range of shared support | Cluster-level density/10 | 9239 / 165 | 0.99 | 0.83-1.18 | 0.868 |
|  |  | Coverage-adjusted cluster-level -level density/10 | 9239 / 165 | 1.01 | 0.82-1.25 | 0.933 |
|  |  | Intact coverage-adjusted cluster-level density/10 | 10104 / 185 | 0.78 | 0.64-0.94 | 0.011 |
|  | ATSB arm only, range of shared support | Cluster-level density/10 | 2686 / 58 | 1.17 | 0.75-1.83 | 0.487 |
|  |  | Coverage adjusted cluster-level density/10 | 2686 / 58 | 1.46 | 0.84-2.51 | 0.176 |
|  |  | Intact coverage-adjusted cluster-level density/10 | 3551 / 78 | 0.61 | 0.44-0.85 | 0.003 |
| Local | Both arms, range of shared support | Local density/10 | 11818 / 213 | 0.98 | 0.96-1.01 | 0.249 |
|  |  | Coverage-adjusted local density/10 | 11818 / 213 | 0.99 | 0.96-1.02 | 0.471 |
|  |  | Intact coverage-adjusted local density/10 | 12081 / 213 | 0.94 | 0.91-0.98 | 0.002 |
|  | ATSB arm only, range of shared support | Local density/10 | 5265 / 106 | 0.98 | 0.96-1.02 | 0.322 |
|  |  | Coverage-adjusted local density/10 | 5265 / 106 | 0.99 | 0.95-1.03 | 0.578 |
|  |  | Intact coverage-adjusted local density/10 | 5526 / 106 | 0.93 | 0.90-0.98 | 0.002 |
| **Models are fitted using rescaled density values which have been divided by 10, effect estimates therefore reflect effect of a 10-unit increase in ATSB density on malaria incidence* | | | | | | |

**Table B**

Summary of secondary outcome indicator coefficient of variation calculated by either Method-of-moments [1], or using a Poisson model with cluster-level random effect and no predictors pooling data from both trial years within each site [2]

| **Outcome** | **Estimate** | **Method-of-moments** | | | **Model-based estimate (95% CI)** | | |
| --- | --- | --- | --- | --- | --- | --- | --- |
|  |  | **Kenya** | **Mali** | **Zambia** | **Kenya** | **Mali** | **Zambia** |
| Prevalence | Protocol | 0.38 | 0.4 | 0.32 | N/A | N/A | N/A |
|  | Baseline | N/A | 0.35 | 0.26 | N/A | 0.34  (0.26-0.45) | 0.13  (0.07-0.25) |
|  | Trial, control arm | 0.49 | 0.39 | 0.32 | 0.50  (0.39-0.64) | 0.23  (0.17-0.33) | 0.22  (0.16-0.31) |
|  | Trial, intervention arm | 0.49 | 0.39 | 0.33 | 0.48  (0.37-0.61) | 0.27  (0.20-0.37) | 0.25  (0.18-0.34) |
| Parity | Protocol | 0.2 | 0.2 | 0.2 | N/A | N/A | N/A |
|  | Trial, control arm | 0.09 | 0.13 | 0.12 | 0.05  (0.02-0.14) | 0.12  (0.08-0.18) | 0.08  (0.04-0.15) |
|  | Trial, intervention arm | 0.07 | 0.12 | 0.11 | 0.04  (0.02-0.09) | 0.11  (0.07-0.16) | 0.04  (0.01-0.14) |
| Abundance | Trial, control arm | 1.14 | 1.47 | 0.73 | 1.06  (0.65-1.75) | 1.35  (0.90-2.03) | 0.67  (0.42-1.07) |
|  | Trial, intervention arm | 1.53 | 0.46 | 0.81 | 1.43  (0.87-2.34) | 0.34  (0.18-0.64) | 0.75  (0.48-1.19) |
| Landing rate | Trial, control arm | 1.31 | 0.86 | 0.8 | 1.22  (0.75-2.01) | 0.78  (0.52-1.18) | 0.73  (0.46-1.17) |
|  | Trial, intervention arm | 2.02 | 0.53 | 1.17 | 1.88  (1.15-3.08) | 0.45  (0.28-0.72) | 1.10  (0.70-1.72) |
| Sporozoite prevalence | Trial, control arm | 0.55 | 0.45 | 0.78 | 0.45  (0.3-0.88) | 0.30  (0.12-0.75) | 0.54  (0.29-1.00) |
|  | Trial, intervention arm | 0.56 | 0.42 | 0.64 | 0.31  (0.12-0.77) | 0.12  (0.001-8.69) | 9.7e^-11^  (1.9e^-16^-5.0e^-5^) |
| *Baseline CV estimates excluded any clusters that were dropped between baseline and trial. Kenya did not conduct a baseline prevalence survey* | | | | | | | |

**References**

1.	Hayes RJ, Moulton LH. Cluster randomised trials. 2nd ed. London: Chapman and Hall; 2017.

2.	Biggs J, Challenger JD, Dee D, Elobolobo E, Chaccour C, Saute F, et al. Characterisation of between-cluster heterogeneity in malaria cluster randomised trials to inform future sample size calculations. Nat Commun. 2025;16(1):6615. doi: 10.1038/s41467-025-61502-w.
